# An Update on Viral Conjunctivitis Treatment Strategies: A Narrative Literature Review

**DOI:** 10.3390/microorganisms13081712

**Published:** 2025-07-22

**Authors:** Maheshver Shunmugam, Francesca Giovannetti, Sonia N. Yeung, Alfonso Iovieno

**Affiliations:** 1 Department of Ophthalmology and Visual Sciences, University of British Columbia, 2550 Willow Street, Vancouver, BC V5Z 3N9, Canada; mahesh14@student.ubc.ca (M.S.); sonia.y@gmail.com (S.N.Y.); 2 Department of Sense Organs, Sapienza University of Rome, 00161 Rome, Italy; francesca.giovannetti@uniroma1.it

**Keywords:** viral conjunctivitis, povidone–iodine, subepithelial infiltrates, adenoviral conjunctivitis, epidemic keratoconjunctivitis

## Abstract

Viral conjunctivitis is a highly contagious ocular condition that significantly impacts patient quality of life and healthcare resources. Despite its self-limiting nature, the condition remains a significant public health concern due to its high transmissibility, prolonged symptoms, and potential complications such as subepithelial infiltrates (SEIs). This review aimed to synthesize and evaluate current management strategies for adenoviral conjunctivitis and provide an evidence-based treatment framework. A systematic literature search of PubMed and the Cochrane Library was conducted, identifying 25 eligible studies published between 2009 and 2024 that focused on clinical interventions including supportive care, antiseptics, corticosteroids, antivirals, and immune modulators. The findings indicate that while supportive therapy and hygiene measures remain central to care, antiseptic agents, specifically povidone–iodine, and topical steroids offer additional benefit in reducing symptom duration and complications. Combination therapies integrating antiseptics, corticosteroids, and immunomodulators show promise for more severe cases, especially those complicated by SEIs. This review proposes an evidence-based comprehensive, multimodal approach management algorithm while highlighting the need for future research in antiviral development and diagnostic innovation to avoid mistreatment and unnecessary antibiotic use.

## 1. Introduction

Conjunctivitis refers to inflammation or infection of the conjunctiva, characterized by hyperemia, edema, and often associated discharge. This term encompasses a wide spectrum of disorders of the ocular surface, mostly self-limiting, although a sub-set can progress to ocular and systemic complications [1].

Conjunctivitis can be categorized by duration (acute, sub-acute, or chronic) and by etiology, including infectious (viral and bacterial) and non-infectious forms (allergic, toxic, inflammatory, and cicatricial) [1].

Accurate identification of the etiology of conjunctivitis is essential for timely triage and appropriate management. Allergic conjunctivitis is characteristically associated with ocular pruritus and is often accompanied by a history of exposure to known allergens such as pollen, animal dander, smoke, or environmental pollutants. Patients frequently report a pattern of seasonal or perennial recurrence, which can aid in diagnosis. During clinical examination, it is imperative to exclude the presence of ocular foreign bodies—including contact lenses, ocular prostheses, retained sutures, or cyanoacrylate glue—which may mimic or exacerbate conjunctival inflammation [2].

Toxic keratoconjunctivitis is often mistaken for allergic conjunctivitis due to overlapping features such as conjunctival hyperemia, chemosis, and a papillary reaction. However, toxic conjunctivitis typically arises from chronic exposure to topical agents, most commonly preservative-containing medications used in the management of ocular hypertension, or from cosmetic products applied to the eyelids [3].

Cicatricial conjunctivitis is defined by conjunctival scarring and is usually seen in the context of ocular trauma, thermal or chemical injury, or autoimmune conditions such as ocular cicatricial pemphigoid [4].

Among infectious causes, bacterial conjunctivitis is commonly caused by Staphylococcus aureus, Streptococcus pneumoniae, and Haemophilus influenzae. Other frequent pathogens include Pseudomonas aeruginosa, Moraxella lacunata, Streptococcus viridans, and Proteus mirabilis. In cases of hyperacute conjunctivitis, clinicians must maintain a high index of suspicion for Neisseria gonorrhoeae and Neisseria meningitidis, given their potential for rapid progression and systemic involvement. Risk factors supporting a bacterial etiology include poor contact lens hygiene, exposure to contaminated materials, crowded living environments, immunocompromised status, and close contact with individuals exhibiting similar symptoms [1].

Viral conjunctivitis, however, accounts for up to 80% of all conjunctivitis cases, presenting with acute-onset redness, irritation, watery discharge, and photophobia [1,5,6]. Viral conjunctivitis is commonly caused by adenoviruses, accounting for approximately 65–90% of cases in adults, with serotypes 3, 4, 7, 8, 19, and 37 most frequently implicated [5,6,7]. Clinical manifestations vary by serotype: epidemic keratoconjunctivitis (serotypes 8, 19, and 37) is characterized by severe conjunctival inflammation, frequent corneal involvement, and a risk of persistent subepithelial infiltrates, whereas pharyngoconjunctival fever (serotypes 3, 4, 7) typically presents in children with high fever, pharyngitis, and bilateral conjunctivitis [8]. Other DNA viruses, including herpes simplex virus (HSV-1) and varicella-zoster virus (VZV), are less common but notable for their distinct presentations: HSV conjunctivitis often appears unilaterally, sometimes accompanied by vesicular periocular lesions and corneal involvement, while VZV may present as part of herpes zoster Ophthalmicus, featuring a dermatomal rash and more severe ocular pain [9]. RNA viruses such as enterovirus 70 and coxsackievirus A24 can cause acute hemorrhagic conjunctivitis, recognizable by sudden onset, prominent subconjunctival hemorrhage, and mild systemic symptoms. Emerging reports have also identified human coronaviruses, Epstein–Barr virus, and others as less frequent etiologies, often associated with mild, self-limited disease [8]. Shared clinical signs include watery discharge, conjunctival injection, eyelid edema, follicular reaction, and preauricular lymphadenopathy [9]. Viral conjunctivitis is highly contagious, spreading through direct contact with secretions or contaminated fomites, and symptoms can persist for up to two weeks [10]. This entity should be suspected in patients with a recent history of viral illness, exposure to contaminated personal items, or clinical signs such as preauricular lymphadenopathy [9,11]. While symptoms often begin in one eye, they typically spread to both. Previous close contact with a person with red eye is also common [12].

Acute infectious conjunctivitis (AIC) is the leading cause of “red eye” consultations seen by family physicians and emergency care providers globally. In the United States, AIC affects more than 6 million individuals annually [12]. Beyond its impact on patient health, the economic burden of AIC is substantial. An estimated USD 430 million dollars is spent on medically unnecessary care associated with AIC of viral etiology alone [13]. Additionally, the highly contagious nature of conjunctivitis often necessitates self-isolation, leading to significant absenteeism from work. These factors underscore conjunctivitis as a threat to both individual health and broader socio-economic stability [1].

Most patients with AIC seek medical attention from general practitioners (GPs) or emergency departments (EDs) [14]. The reliance on non-ophthalmologist care increases the risk of mismanagement, including medically unnecessary antibiotic prescriptions and inappropriate referrals to emergency departments or ophthalmologists. A study by Shekhawat et al. analyzed data from over 340,000 individuals in the US who were newly diagnosed with AIC [15,16]. Notably, they found that nearly 60% of patients filled at least one prescription for topical antibiotics and prescribing patterns varied depending on the healthcare provider [11]. As expected, ophthalmologists were less likely to prescribe antibiotics than urgent care providers, GPs, and pediatricians [15].

The diagnosis of viral conjunctivitis is primarily clinical; however, clinicians may rely on several supplementary rapid diagnostic tests (e.g., AdenoPlus) which offer moderate sensitivity (40–63%) and high specificity, making them practical in clinical settings. That said, these tests require further validation to confirm their reliability [17,18]. The gold standard test for detecting adenoviral DNA due to its high sensitivity and specificity remains polymerase chain reaction (PCR). While PCR is highly accurate, it may have limited accessibility in low-resource settings [19].

Though self-limiting, viral conjunctivitis can disrupt daily activities and cause substantial discomfort. In severe cases, complications such as multifocal subepithelial infiltrates (SEIs), corneal scarring, conjunctival membrane formation, and cicatrizing conjunctivitis with symblepharon may develop, significantly affecting vision and quality of life [19].

Despite the significant body of research analyzing multiple therapeutic options for AIC, there is a generalized lack of implementation of evidence-based strategies in this context. This paper aims to provide a comprehensive assessment of the existing evidence while offering practical, evidence-based recommendations to guide clinicians in the effective management of viral conjunctivitis.

## 2. Methods

A comprehensive literature review was conducted to evaluate current strategies for the management of viral conjunctivitis A systematic search was performed using PubMed and the Cochrane Library employing broad key terms such as “viral conjunctivitis treatment”, “viral conjunctivitis management”, and “viral conjunctivitis”. No language restrictions were initially applied to ensure comprehensive coverage of the literature. Only studies published in English or with available English translations were ultimately included in the final analysis.

The inclusion criteria comprised articles published between 1 January 2009, and 1 January 2024, focusing specifically on the clinical management of viral conjunctivitis. This included studies evaluating supportive care, antiseptic agents, antiviral therapies, corticosteroids, and immune modulators. Studies were excluded if they were centered on epidemiology or pathophysiology, were editorials, commentaries, or responses to the editor, were not peer-reviewed, did not include human subjects, or were not available in English.

**Figure 1:**
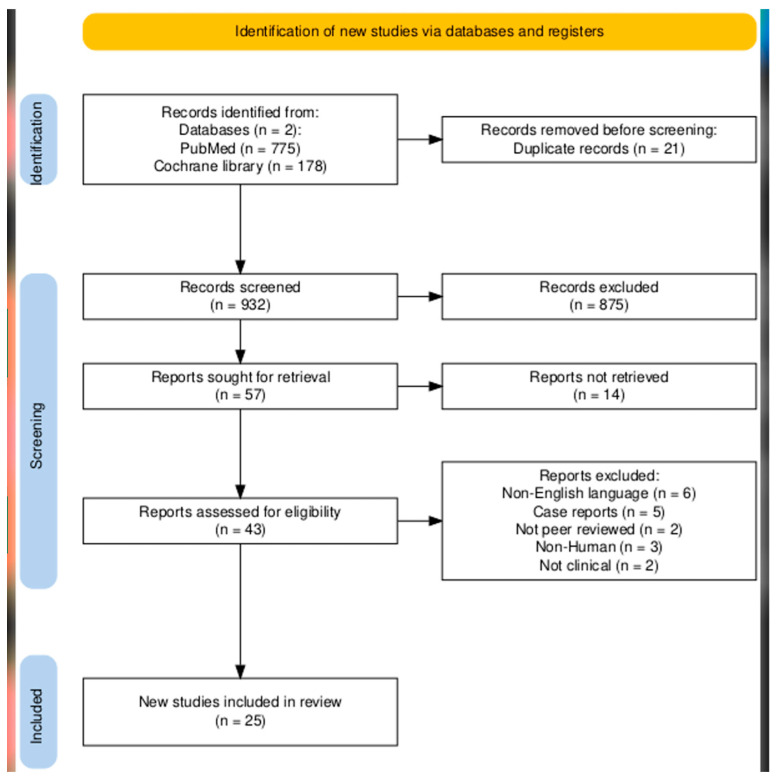
A PRISMA-style flow diagram illustrating the literature selection process for this review. A total of 953 articles were identified from PubMed and the Cochrane Library. After screening and eligibility assessment, 25 studies were included in the final narrative analysis [20].

The selection process involved a detailed screening of titles, abstracts, and references. Priority was given to studies providing high-quality, evidence-based information. A total of 775 articles were screened from PubMed, of which 28 met the inclusion criteria. Additionally, 178 articles were screened from the Cochrane Library, with 10 ultimately included. After excluding duplicates, 25 articles were retained for the final analysis A summary of key studies, their interventions, patient populations, and outcome measures can be found in Appendix A. The earliest included study was published in 2009, and the most recent study included in our paper was published in 2024 (Figure 1).

**Figure 2:**
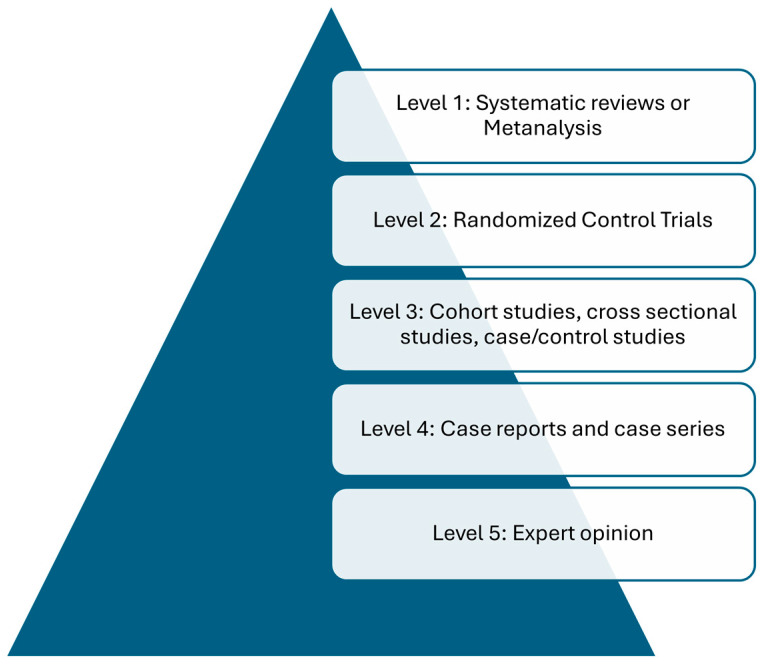
Visual depiction of the “Centre for Evidence-Based Medicine” framework for levels of evidence.

The evidence levels for each intervention were assessed and graded independently by authors M.S. and F.G based on the “Centre for Evidence-Based Medicine” framework (Figure 2). No discrepancies in evidence level grading were found.

## 3. Current Management Approaches

### 3.1. Supportive Therapies

Supportive care (including artificial tears, cold compresses, and appropriate hygiene measures) is, at present, the cornerstone of viral conjunctivitis management. These interventions focus on symptom relief and reducing transmission risk but do not alter the natural course of the disease (Level 1) [21]. Several studies have underscored the importance of supportive measures in treatment. The evidence provided by Liu et al. emphasized the role of combining artificial tears with patient education on hygiene to reduce the spread of infection [21]. Their findings indicated that patients who adhered to these measures and who experienced spontaneous resolution had similar long-term outcomes to those who had managed their AIC with topical medications. They also found that there was evidence to suggest that artificial tears alone were less effective in preventing complications like SEIs.

Further highlighting the value of non-pharmacological approaches, Than et al. demonstrated no significant difference in clinical signs and patient symptoms at final follow-up for AIC treated with supportive care versus single-use topical treatment of 5% povidone–iodine (Level 2) [22]. Indeed, symptoms and signs were significantly reduced on the first visit (day 4) in the treatment cohort, suggesting that treatment may play a role in patient comfort in early disease.

In line with these findings, Azari et al. recommended supportive measures as first-line treatment for most cases due to the self-limiting nature of viral conjunctivitis (Level 1) [17]. Their study supports the understanding that, for most cases, conservative management is sufficient and effective in ensuring recovery. They went on to recommend frequent hand washing, meticulous disinfection of medical instruments, and isolation of AIC patients in the healthcare provider’s office to reduce the risk of local transmission.

Finally, a systematic review of clinical practice guidelines (CPGs) by Chan et al. found that CPGs “consistently recommended non-pharmacological interventions (artificial tears, cold compress, avoidance or removal of allergens) for non-infectious conjunctivitis” (Level 1) [23].

### 3.2. Antiseptics

Povidone–iodine (PVP-I) is a broad-spectrum antiseptic widely used in ophthalmology for preoperative disinfection and infection control. By gradually releasing free iodine, it exerts potent bactericidal, fungicidal, and viricidal effects [24]. Although some authors speculate that PVP-I may be less effective against intracellular adenoviral particles in infected cells in vitro, multiple studies have validated the efficacy of PVP-I [25]. Yates et al. demonstrated rapid virucidal activity at three different concentrations within 1–5 min for most ocular adenovirus types, while longer exposure (15–60 min) was required for others, suggesting that its antiviral efficacy may be adenovirus type-dependent [26]. There is also evidence to suggest that PVP-I is effective in symptom resolution and SEI prevention, though its application requires some caution due to potential ocular surface toxicity and conjunctival goblet cell loss [27].

A one-time in-office conjunctival irrigation with PVP-I has been suggested to be safe and effective in controlling disease severity and complications in a handful of studies. In the RAPID study, Shorter et al. evaluated the safety and tolerability of a single instillation of 5% PVP-I ophthalmic solution compared to artificial tears for the treatment of adenoviral conjunctivitis [28]. The study confirmed that a one-time application of 5% PVP-I was well tolerated, with no reported adverse effects (Level 2) [28]. Similarly, Than et al. investigated the efficacy and safety of a single in-office instillation of 5% PVP-I, demonstrating a significant reduction in PCR viral titers and lower severity scores on day 4 (Level 2) [22]. Vats et al. explored the use of a single administration PVP-I lower-concentration (1%) wash in combination with 0.5% Moxifloxacin and artificial tears. This treatment combination reduced clinical signs, patient symptom time, and the development of SEI significantly when compared to antibiotic and artificial tear use alone (Level 2) [29].

In a pediatric population, Ozen et al. investigated the impact of a one-time in-office conjunctival irrigation with 2.5% PVP-I in infants with adenoviral conjunctivitis, finding that treated patients exhibited lower clinical severity scores and significantly faster recovery (on average, 7 days) compared to the control group (Level 2) [30].

Bekmez et al. in a subsequent study confirmed that a 2.5% PVP-I one-time irrigation in pediatric patients significantly shortened recovery time from 12 to 7 days in comparison to artificial tears without notable adverse effects (Level 3) [31].

The efficacy of a short course of PVP-I in reducing symptom duration has been studied for different concentrations. Yazar et al. demonstrated that administering 0.5% PVP-I three times daily for two weeks shortened recovery time compared to controls (Level 3) [32]. Altan-Yaycioglu et al. emphasized the long-term benefits of diluted 2% PVP-I irrigation, demonstrating a significant reduction in the development of complications such as SEIs in their treatment group (Level 2) [33]. Similarly, Trinavarat et al. evaluated the effects of 2% PVP-I four times daily for seven days and reported a 77% recovery rate within the first week (Level 3) [34].

PVP-I has also been investigated in combination with dexamethasone. After proving its safety and efficacy in a rabbit model of adenoviral conjunctivitis, various concentrations of PVP-I in combination with 0.1% dexamethasone have been studied [35]. In a pilot study in 2009, a 0.4% PVP-I concentration in combination with 0.1% dexamethasone demonstrated clinical and infection resolution by day 5 (Level 3) [36]. In a randomized clinical trial, Pepose al. evaluated treatment with 0.6% PVP-I/0.1% dexamethasone vs. vehicle four times daily for five days in patients with positive a Rapid Pathogen Screening Adeno-Detector Plus test [37]. Their study suggested that their combination was safe and considerably improved clinical signs by day 6, though this did not reach statistical significance (Level 2) [37]. A significant reduction in the duration of conjunctivitis was confirmed in another randomized clinical trial by Pinto et al. but there was no statistically significant difference between the treatment group with 0.4% PVP-I/0.1% dexamethasone four times daily for seven days and the artificial tears control group in reference to the patients’ intraocular pressures and the development of SEIs (Level 2) [38]. In a systematic review and network metanalysis by Chen et al., combination therapy with 1% PVP-I and 0.1% dexamethasone demonstrated a trend toward reducing all clinical signs compared to placebo, though the results did not reach statistical significance (Level 1) [39]. Kovalyuk et al. studied the efficacy of a seven-day treatment with 1% PVP-I/0.1% dexamethasone four times a day in comparison to 0.1% dexamethasone alone and artificial tears alone in a randomized clinical trial [40]. The combination therapy was significantly effective in expediting recovery and resulted in a superior reduction in symptoms (such as itching and tearing) and signs (such as conjunctival injection, superficial punctuate keratopathy, and pseudomembranes) (Level 2) [40].

### 3.3. Corticosteroids

Corticosteroids are widely used for symptom control in adenoviral conjunctivitis, particularly in cases complicated by significant inflammation.

As previously described, topical steroids have been studied in combination with antiseptics with proven efficacy in managing viral conjunctivitis. The concerns regarding corticosteroid use, such as increased IOP, prolonged viral shedding, and HSV reactivation, are not strongly supported by high-quality evidence, particularly in cases of short-term use [41]. In fact, literature reviews suggest that topical corticosteroids are effective and well tolerated when used for brief periods, especially in combination with antibiotics, antiseptics, or anti-infectives [42]. A 2011 study compared the effects of dexamethasone 0.1% and hypromellose 0.3% four times a day for one week in patients with acute presumed viral conjunctivitis [36]. The results showed that more patients felt that the dexamethasone treatment helped, with no significant differences in discomfort or physician-assessed conjunctival hyperemia, supporting the use of short-term dexamethasone for such cases without harmful effects (Level 2) [43].

Santiago et al. suggested that use of prednisolone 1% for 15 days reduced AIC symptoms in comparison to artificial tears at the first follow-up but failed to show significant differences between symptoms and signs at the final follow-up (Level 2) [44]. Kocluk et al. concluded that both loteprednol and dexamethasone had comparable efficacy for symptom control. It is worth noting that while dexamethasone showed non-significant evidence in relation to reducing symptoms faster, loteprednol use had fewer IOP-related adverse effects (Level 3) [45]. Therefore, for patients at risk of intraocular pressure (IOP) elevation, the authors suggest that loteprednol is considered a safer alternative [45].

The authors highlight that corticosteroids are crucial in managing SEIs in viral conjunctivitis.

Dexamethasone and loteprednol were compared in the study by Kolcuk et al. involving patients who developed bilateral SEIs. Eyes with dexamethasone administration had faster symptom relief, though this was not statistically significant, and both groups reported similar levels of recurrence (Level 3) [45].

A study by Gouider et al. comparing 0.5% cyclosporine A (CSA) and fluorometholone (FML) for subepithelial infiltrates (SEIs) in epidemic keratoconjunctivitis found that FML resulted in significantly faster resolution at 3 and 6 months, while CSA showed significantly less recurrence of SEIs after 6 months [46]. Both treatments were suggested to be equally safe and tolerated (Level 2) [46].

In a study by Matsuura et al., patients were divided into groups receiving 1.5% levofloxacin with 0.1% FML administered four times a day versus 0.1% PVP-I with 0.1% FML four times a day. They found that the acute signs and symptoms as well as the viral load had no significant differences between the groups; however, after 15 days, the proportion of patients with SEIs treated with PVP-I was significantly lower (Level 2) [47].

When corticosteroids are insufficient or contraindicated, immune modulators like tacrolimus and cyclosporine A are proposed as effective in managing persistent or recurring SEIs. Corticosteroid treatment of SEIs can result in a 17.5% recurrence rate, with subsequent difficulties with tapering the medication successfully [48]. Therefore, in a 2010 study, topical 1% cyclosporine A (CsA) was evaluated as an alternative treatment for SEIs in patients unresponsive to or unable to tolerate corticosteroids [49]. The study suggested that CsA was both safe and effective, reducing medication use and symptom severity while improving patient satisfaction (Level 3) [49]. These findings were duplicated in a 2012 randomized clinical study, where topical 0.05% cyclosporine A was also suggested to be a safe and effective alternative to treat SEIs in patients unresponsive to corticosteroids or with high IOP due to their use, significantly improving BCVA, reducing infiltrates, and lowering IOP (Level 2) [50]. In a mean follow-up period of nine months, most patients experienced clinical improvement with minimal side effects, though two eyes out of sixteen showed recurrence after 3 months of treatment [50].

Similarly, topical 0.03% tacrolimus ointment was suggested to be a safe and effective alternative to treat SEIs in patients unresponsive to or intolerant to corticosteroids, significantly improving CDVA and symptom severity [51]. However, three out of eleven patients discontinued treatment due to side effects such as dizziness [50]. These results are compatible with another study by Ghanem et al., who concluded that topical 0.03% tacrolimus was indeed effective in reducing SEIs and improving CDVA in patients with corticosteroid-resistant SEIs after adenoviral keratoconjunctivitis, with significant symptom relief and no IOP elevation (Level 3) [51]. One patient out of seven could not tolerate the medication, but overall, it proved to be a viable corticosteroid-sparing option [51]. In a study by Bhargava et al., tacrolimus 0.03% was suggested to be as effective as dexamethasone in reducing SEIs, though tacrolimus carried a much lower risk of subsequent IOP elevation (Level 2) [50].

### 3.4. Antivirals

Currently, antiviral agents such as acyclovir, trifluridine, and valacyclovir are widely used for herpes virus infections, while treatment options for adenoviral conjunctivitis remain limited [52]. Although cidofovir has shown some efficacy in in vivo studies of adenoviral conjunctivitis, its use is restricted due to its associated toxicity and cost [53,54]. Other agents, like idoxuridine, have also largely been abandoned for their high ocular toxicity [55]. While the growing number of antiviral drugs is promising, their efficacy in treating viral conjunctivitis remains uncertain, and many carry a risk of adverse effects. Notably, most antiviral drug development has focused on herpes virus infections, with limited progress in targeted therapies for adenoviral conjunctivitis. The 2022 systematic review with metanalysis conducted by Liu et al. concluded that the notion of antiviral agents shortening the duration of symptoms or signs when compared with artificial tears was not statistically proven (Level 1) [21].

Cidofovir has been studied in rabbit models of viral conjunctivitis in multiple studies. Topical cidofovir reduced adenoviral titers and shedding duration, but resistant viral strains emerged [41,53]. Clinical trials on topical cidofovir for adenoviral keratoconjunctivitis, with and without cyclosporine, showed no significant benefit in symptom resolution, though higher doses reduced severe corneal opacities but caused local adverse effects [54,55].

Ganciclovir 0.15% may be used together with 2.5% PVP-I irrigation in children with adenoviral conjunctivitis, potentially helping speed up improvement (Level 3) [31].

Trifluridine has been used to treat conjunctivitis caused by adenoviruses, herpesviruses, and smallpox, with mixed results. While some cases showed improvement, clinical trials did not demonstrate significant benefits over other treatments, leaving its efficacy uncertain [55].

Clinical studies on Interferon Beta for viral conjunctivitis show promising results, reporting reduced disease duration and SEI occurrence [55,56]. Further studies are needed to explore this treatment.

Beyond currently available treatments, several investigational agents show promise in the management of adenoviral conjunctivitis. Ranpirnase (OKG-0301), a novel ribonuclease which inhibits protein synthesis by degrading tRNA, demonstrated potent antiviral activity in infected Ad5 rabbit models, reducing viral titers and infection duration [57]. These findings are comparable to a study employing 0.5% cidofovir [58]. In another study, cyclopentenylcytosine (CPE-C), a nucleoside analog with broad-spectrum antiviral activity, exhibited low effective concentrations against multiple ocular adenovirus serotypes in vitro and showed comparable in vivo efficacy to cidofovir without inducing ocular irritation [59]. Additionally, non-specific immunomodulatory strategies are being explored. A nanoparticle-based formulation containing dual TLR3/9 agonists has demonstrated efficacy in feline herpesvirus keratitis, significantly reducing symptoms and viral shedding without adverse effects [54]. Although these therapies remain in early investigational phases, they highlight promising directions in the development of targeted, tolerable, and effective antiviral treatments for adenoviral ocular infections.

### 3.5. Proposed Management Plan

Together, these findings help unravel the treatment strategies for adenoviral conjunctivitis, emphasizing the need for a tailored approach based on disease severity and patient-specific risk factors. While supportive care remains the mainstay of management, the integration of antiseptics, corticosteroids, and immune modulators offers additional benefits in improving outcomes and reducing long-term sequelae.

**Figure 3:**
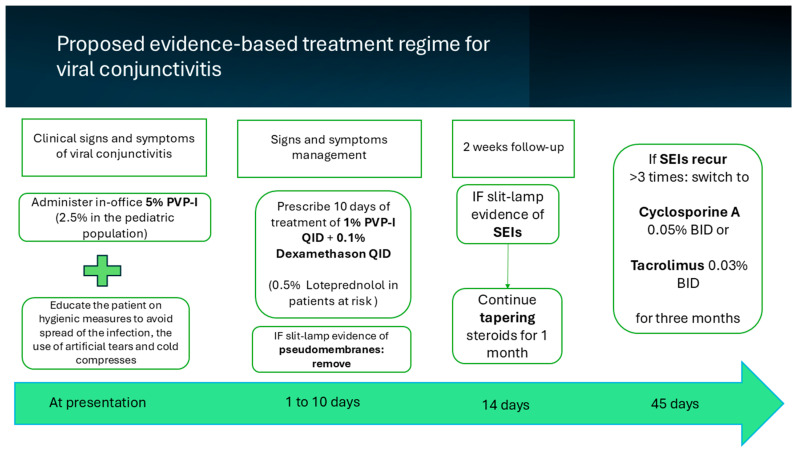
The proposed evidence-based treatment plan for managing viral conjunctivitis.

Based on the latest evidence discussed in Section 3, the following therapeutic plan is proposed for managing viral conjunctivitis, and it is presented graphically in Figure 3.

#### 3.5.1. Management at Presentation

The approach begins with an in-office administration of antiseptic treatment with PVP-I. For infants, children, and pregnant women, a lower concentration of 2.5% PVP-I may be recommended. For all other patients, PVP-I at concentrations ranging from 2.5% to 5% should be considered based on availability, patient age, and tolerability. We emphasize that the administration of PVP-I for a patient with a dry ocular surface should be considered on a case-by-case basis. Clinicians should also focus on patient education, emphasizing the use of artificial tears, cold compresses to alleviate symptoms, and strict hygiene measures to reduce transmission.

#### 3.5.2. Proposed Treatment Regimen and Follow-Up

Following the one-time, in-office PVP-I wash, patients should be prescribed topical povidone–iodine 1%, four times daily for 7 to 10 days, in combination with topical corticosteroids such as dexamethasone 0.1%, loteprednol, or fluorometholone (FML), also four times daily for a similar duration.

A follow-up visit after two weeks is essential to reassess disease progression and guide further therapy. When SEIs are seen on biomicroscopy examination, the topical steroids should be slowly tapered, reducing the dosage by one drop every week for a total of four weeks.

#### 3.5.3. Management of Complications

For persistent or recurrent SEIs, e.g., more than three recurrences, in cases where corticosteroid side effects occur, such as elevated intraocular pressure, or in patients with steroid-refractory SEIs, immunomodulatory agents like cyclosporine A 0.05% drops two times a day or topical tacrolimus 0.03% ointment two times a day may be introduced as safe and effective alternatives.

In severe cases of adenoviral keratoconjunctivitis, an intense inflammatory response can lead to pseudomembrane formation. In-office removal of the pseudomembrane is recommended to reduce discomfort and prevent complications such as conjunctival fibrosis and long-term sequelae [17]. The presence of pseudomembranes alongside SEIs underscores the need for topical anti-inflammatory treatment, with steroids or immunomodulators being crucial in managing the inflammation [50].

## 4. Conclusions

Accurate differentiation of conjunctivitis etiologies is essential to reduce unnecessary antibiotic prescriptions, support antimicrobial stewardship, and ensure appropriate management and infection control. Allergic conjunctivitis typically presents with bilateral itching, chemosis, and a history of allergen exposure or seasonal recurrence. Bacterial conjunctivitis is often characterized by purulent discharge and eyelid crusting and may be associated with poor hygiene, contact lens use, or close contact with infected individuals; hyperacute cases raise concern for *Neisseria* species. Viral conjunctivitis frequently follows an upper respiratory infection and presents with watery discharge, follicular conjunctival reaction, and preauricular lymphadenopathy. Toxic conjunctivitis results from chronic exposure to topical medications or cosmetics and mimics allergic forms but lacks an atopic history. Cicatricial conjunctivitis, marked by conjunctival scarring, suggests autoimmune, traumatic, or chemical causes. A thorough history and targeted clinical examination are critical to distinguishing among these presentations.

Viral conjunctivitis remains a significant public health concern due to its high transmissibility, prolonged symptomatology, and risk of complications such as SEIs. While the condition is often self-limiting, optimizing management strategies is crucial to improving patient outcomes and minimizing social impact.

Current approaches rely on supportive care and antiseptic agents like povidone–iodine to reduce symptom duration and prevent complications. However, the absence of targeted antiviral therapies and the limited accessibility of rapid diagnostic tools continue to pose challenges and lead to mistreatment. Enhancing diagnostic accuracy through cost-effective and widely available assays, such as improved PCR-based techniques, could facilitate early intervention, reduce unnecessary antibiotic use, and streamline patient management.

Future research should prioritize the development of effective antiviral agents and explore combination therapies that integrate antiseptics, antivirals, and immune modulators to provide both symptomatic relief and long-term disease control.

By refining current protocols and advancing therapeutic innovations with a comprehensive, evidence-based management strategy, we can enhance patient care. The proposed management algorithm may prove useful for ophthalmologists and other medical doctors treating AIC. Future research should focus on refining therapeutic options and developing robust protocols for rapid diagnosis and treatment.

## Data Availability

No new data were created or analyzed in this study. Data sharing is not applicable to this article.

## Appendix A

**Table A1:** Summary of key studies, their interventions, patient populations, and outcome measures.

Study Title	Study Design	Patient Population	Interventions	Outcome Measures
Efficacy of povidone-iodine-containing therapies for treatment of adenoviral conjunctivitis: A systematic review and network meta-analysis. [37]	Systematic review and network metanalysis	A total of 5 articles were included, published between 2015 and 2019	PVP-I	Clinical efficacy of treatment
Topical Pharmacological interventions versus placebo for epidemic keratoconjunctivitis [19]	Cochrane systematic review	A total of 10 articles were included, published between 1980 and 2021	Varied	Clinical diagnosis and treatment strategies
A systematic review of clinical practice guidelines for infectious and non-infectious conjunctivitis [21]	Systematic review	A total of 15 articles were included, published between 2015 and 2019	Varied	Clinical practice guidelines for conjunctivitis treatment
Conjunctivitis: A systematic review [11]	Systematic review	A total of 167 articles were included, published between 1964 and 2020	Varied	Clinical diagnosis and treatment strategies
Comparison of the safety and efficacy of topical Tacrolimus (0.03%) versus dexamethasone (0.05%) for subepithelial infiltrates after adenoviral conjunctivitis [46]	Double-masked randomized control trial	90 adults in 2019	Treatment arm 1: tacrolimus 0.03% Treatment arm 2: dexamethasone 0.05%	Incidence of subepithelial infiltrates
Safety and tolerability of a one-time, in-office administration of 5% povidone-iodine in the treatment of adenoviral conjunctivitis: The Reducing Adenoviral Patient Infected Days (RAPID) study [26]	Double-masked randomized control trial	56 adults, mean age of 34, between 2015 and 2018	Treatment arm 1: PVP-I 5% Control: preservative-free artificial tears	Corneal fluorescein staining, visual acuity, and participant rated overall ocular discomfort
Comparative study on the efficacy of non-steroidal, steroid and non-use of anti-inflammatory in the treatment of acute epidemic conjunctivitis [42]	Prospective double-masked randomized control study	37 adults	Treatment arm 1: prednisolone 1%/ciprofloxacin 0.3% Treatment arm 2: sodium diclofenac 0.1%/ciprofloxacin 0.3% Control: ciprofloxacin 0.3%/artificial tears	Clinical signs and symptoms
A randomized controlled phase 2 trial of povidone-iodine/dexamethasone ophthalmic suspension for acute viral conjunctivitis [41]	Randomized, double-masked, parallel-group, vehicle-controlled study	132 adults, mean age of 31, between 2013 and 2014	Treatment arm 1: PVP-1 0.6%/dexamethasone 0.1% Control: vehicle	Clinical resolution of acute viral conjunctivitis in the study eye
Efficacy of a Single Administration of 5% Povidone-Iodine in the Treatment of Adenoviral Conjunctivitis [20]	Prospective double-masked, pilot, randomized trial	56 adults, mean age of 39, between 2015 and 2018	Treatment arm 1: PVP-I 5% Control: artificial tears	Percent reduction from peak viral load; clinical signs and symptoms
Treatment of adenoviral keratoconjunctivitis with a combination of povidone-iodine 1.0% and dexamethasone 0.1% drops: a clinical prospective controlled randomized study [38]	Prospective, randomized, controlled, double-blinded clinical trial	68 adults, 78 eyes, mean age of 47	Treatment arm 1: PVP-I 1.0%/dexamethasone 0.1% Treatment arm 2: dexamethasone 0.1% Control: Hypromellose 0.3%	Rate of improvement in symptoms and signs of adenoviral keratoconjunctivitis in treatment groups
One-time low concentration betadine eye wash: A novel treatment for epidemic viral conjunctivitis for accelerated recovery [27]	Prospective, double-masked, randomized control study	1328 adults, mean age of 29, in 2023	Treatment arm 1: betadine 1%/moxifloxacin 0.5%/artificial tears Control: moxifloxacin 0.5%/artificial tears	Conjunctival signs of inflammation, patient satisfaction level, and symptom reduction
Corticosteroids Versus Cyclosporine for Subepithelial Infiltrates Secondary to Epidemic Keratoconjunctivitis: A Prospective Randomized Double-Blind Study [44]	Prospective, double-blind randomized control study	51 adults and 72 eyes, mean age of 36, between 2017 and 2019	Treatment arm 1: fluorometholone 0.1% Control: cyclosporin 0.5%	SEI reduction and visual acuity improvement
Dexamethasone/Povidone Eye Drops versus Artificial Tears for Treatment of Presumed Viral Conjunctivitis: A Randomized Clinical Trial [41]	Randomized, masked, and controlled trial	122 adults, mean age of 35, between 2011 and 2012	Treatment arm 1: dexamethasone 0.1%/povidone–iodine 0.4% Control: artificial tears	Disease duration, overall discomfort, itching, foreign body sensation, tearing, redness, eyelid swelling, side effects of the eye drops, intraocular pressure, and the incidence of subepithelial corneal infiltrates
Symptomatic Treatment of Subepithelial Infiltrates after Viral Conjunctivitis: Loteprednol or Dexamethasone? [43]	Unmasked prospective, comparative clinical study	15 adults, 30 eyes, between 2014 and 2015	Eye 1: loteprednol Eye 2: dexamethasone 0.1%	SEI reduction and visual acuity improvement
Povidone iodine in the treatment of adenoviral conjunctivitis in infants [28]	Prospective, unmasked, clinical trial	35 infants, 35 eyes, mean age of 3 months, in 2012	Treatment arm 1: PVP-I 2.5%/artificial tears Control: artificial tears	Lid edema, conjunctival chemosis, fragility of conjunctival vasculature, pseudomembrane formation, and corneal involvement were scored clinically
Comparative study of topical regimen for adenoviral keratoconjunctivitis by 0.1% fluorometholone with and without polyvinyl alcohol iodine [45]	Prospective open-label study	19 adults, 27 eyes, mean age of 53, between 2017 and 2019	Treatment arm 1: 1.5% levofloxacin/0.1% fluorometholone Treatment arm 2: diluted PVP-I/0.1% fluorometholone	Acute signs and symptoms, HAdV DNA copy number, and the presence of multiple subepithelial corneal infiltrates
Effect of diluted povidone iodine in adenoviral keratoconjunctivitis on the rate of subepithelial corneal infiltrates [31]	Retrospective chart review	211 adults, mean age od 33, between 2013 and 2016	PVP-I 2% compared to all other medications	Incidence of complications and sequelae from adenoviral conjunctivitis
The treatment Models for Adenoviral Keratoconjunctivitis in the Childhood Population [29]	Retrospective chart review	50 children, mean age of 13, between 2016 and 2019	Treatment arm 1: PVP-I 2.5%/ganciclovir 0.15%/sodium hyaluronate 0.15% Treatment arm 2: PVP-I 2.5%/sodium hyaluronate 0.15% Treatment arm 3: ganciclovir 0.15%/sodium hyaluronate 0.15%	Clinical findings
The effects of povidone iodine (pH 4.2) on patients with adenoviral conjunctivitis. [30]	Retrospective chart review	112 adults, between 2011 and 2014	Treatment arm 1: PVPI-0.5% Control: natural tears	Clinical findings and side effects
Treatment of epidemic keratoconjunctivitis with 2% povidone-iodine: a pilot study. [32]	Prospective, interventional, uncontrolled study	61 adults, 2012	Treatment arm 1: PVP-I 2%	Recovery rate within 1 week, drug tolerability, and sequelae
A combination povidone-iodine 0.4%/dexamethasone 0.1% ophthalmic suspension in the treatment of adenoviral conjunctivitis. [35]	Prospective, open-label, single-armed, phase II clinical trial in humans	6 adults, 9 eyes, in 2008	Treatment arm 1: PVP-I 0.4%/dexamethasone 0.1%	Clinical findings and viral titers
A randomized placebo-controlled trial of topical steroid in presumed viral conjunctivitis. [40]	Randomized control trial	88 adults, between 2003 and 2007	Treatment arm 1: dexamethasone 0.1% Control: Hypromellose 0.3%	Patient comfort and conjunctival hyperemia
Topical treatment with 1% cyclosporine for subepithelial infiltrates secondary to adenoviral keratoconjunctivitis. [47]	Prospective, uncontrolled trial	4 adults, 8 eyes, mean age of 45, in 2010	Treatment arm 1: CsA 0.05%	Best-corrected decimal visual acuity, intraocular pressure, and evaluation of severity of SEIs
Cyclosporine a 0.05% eye drops for the treatment of subepithelial infiltrates after epidemic keratoconjunctivitis. [48]	Prospective double-blind randomized study	28 adults, 34 eyes, mean age of 36, in 2020	Treatment arm 1: 0.05% CsA Treatment arm 2: FML	Clinical findings and SEI prevalence
Tacrolimus for the treatment of subepithelial infiltrates resistant to topical steroids after adenoviral keratoconjunctivitis. [49]	Prospective, nonrandomized, noncomparative interventional case series	7 adults, 10 eyes, mean age of 37, between 2009 and 2013	Treatment arm 1: 0.03% tacrolimus	Age, sex, CDVA, intraocular pressure, duration and intensity of symptoms, biomicroscopy findings, and duration of therapy
